# Successful bilateral pudendal neuromodulation to treat male detrusor areflexia following severe pubic symphysis fracture, a case report

**DOI:** 10.1186/s12894-015-0108-4

**Published:** 2015-11-18

**Authors:** Serge P. Marinkovic, Brandi Miller, Scott Hughes, Christina Marinkovic, Lisa Gillen

**Affiliations:** Department of Urology, Detroit Medical Center, Harper Hospital, Detroit, MI 48202 USA

**Keywords:** Sacral neuromodulation, Male urinary retention, Pudendal neuromodulation, Unilateral ischial fracture

## Abstract

**Background:**

A Drum Dock Manager in an auto manufacturing company suffers a pelvic fracture, severing the bulbar urethra and completely fracturing the right side of his pelvis.

He is unable to void without catheterization but has a complete sensation to void. Can neuromodulation help him achieve spontaneous voiding?

**Case presentation:**

We reviewed the electronic medical record of Mr. M.E. from Detroit Medical Center following his 2012 forklift accident and subsequent orthopedic surgeries. He successfully underwent bilateral sacral neuromodulation, with a resulting max flow of 16.8 mls/sec and post-void residual urine of 50–100 mls. Unfortunately, he later presented with bilateral pocket and sacral lead infection, and both systems had to be removed. Six weeks later, M.E. had bilateral pudendal neurostimulation placement to avoid the previously infected areas. Max flow improved to 14.5 mls/sec and 0–50 mls residual urine. However, urodynamics proved that his P_det_ at max flow was in excess of 120 cm of H20 pressure while he had been on finesteride and tamsulosin for the preceding five years for the management of his documented benign prostate hyperplasia symptoms. He underwent Green light laser transurethral resection of the prostate and had max flow improvement to 22.5 mls/second with zero residual urine with multiple straight catheterization confirmations.

**Conclusion:**

Sacral neuromodulation may successfully correct traumatic urinary retention in male patients. Additionally, pudendal neuromodulation can be successfully utilized as a salvage method for an infected sacral neuromodulation impulse generator (IPG) and tined lead with a return to proper voiding.

## Background

Traumatic urethral injury may result in partial or total urinary retention in male patients. Clean intermittent catheterization or chronic indwelling Foley catheterization are frequently instituted to properly empty the bladder as a temporary or permanent treatment measure. However, after primary healing of 6 months or more, consideration may be given to the surgical treatment of urinary retention [1]. In the literature, sacral neuromodulation has reportedly been utilized following surgical trauma but not blunt or penetrating trauma. We now report the first case in the literature utilizing bilateral sacral neuromodulation followed by bilateral pudendal neuromodulation after an industrial accident left our male patient with a severe pubic symphysis fracture and subsequent urinary retention.

## Case presentation

**Fig. 1:**
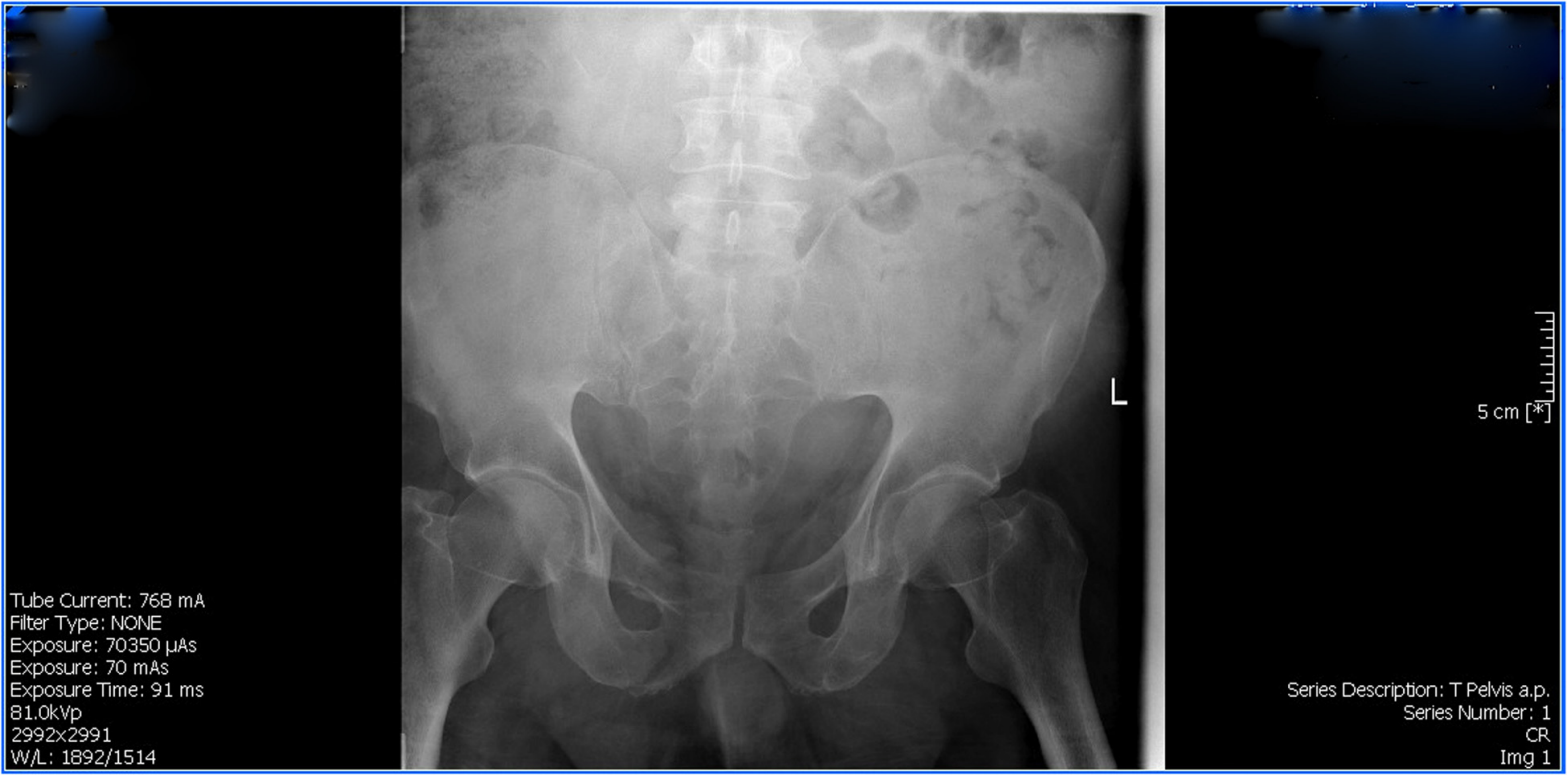
This plain film illustrates a right ischial pelvic fracture

**Fig. 2:**
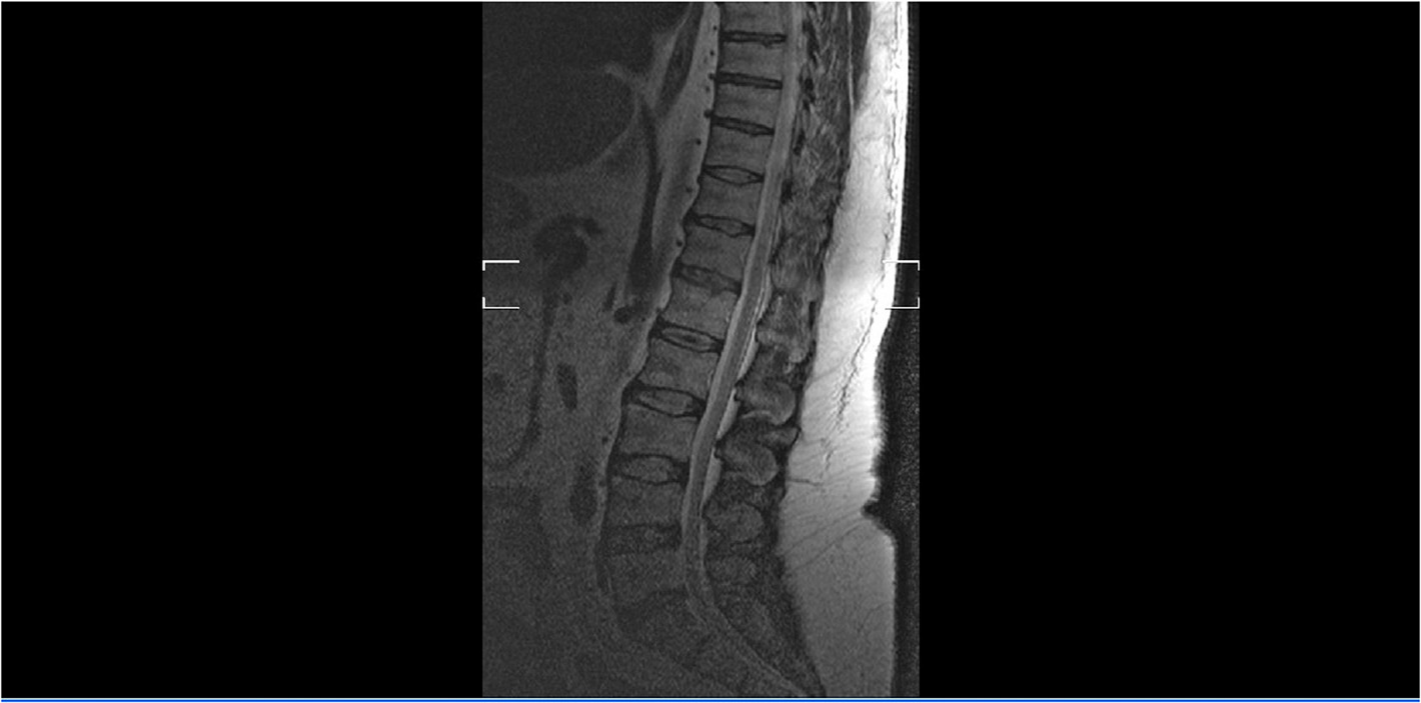
This lateral lumbar Magnetic Resonance Image demonstrates severe degenerative disc disease with a few bulging intervertebral discs

**Fig. 3:**
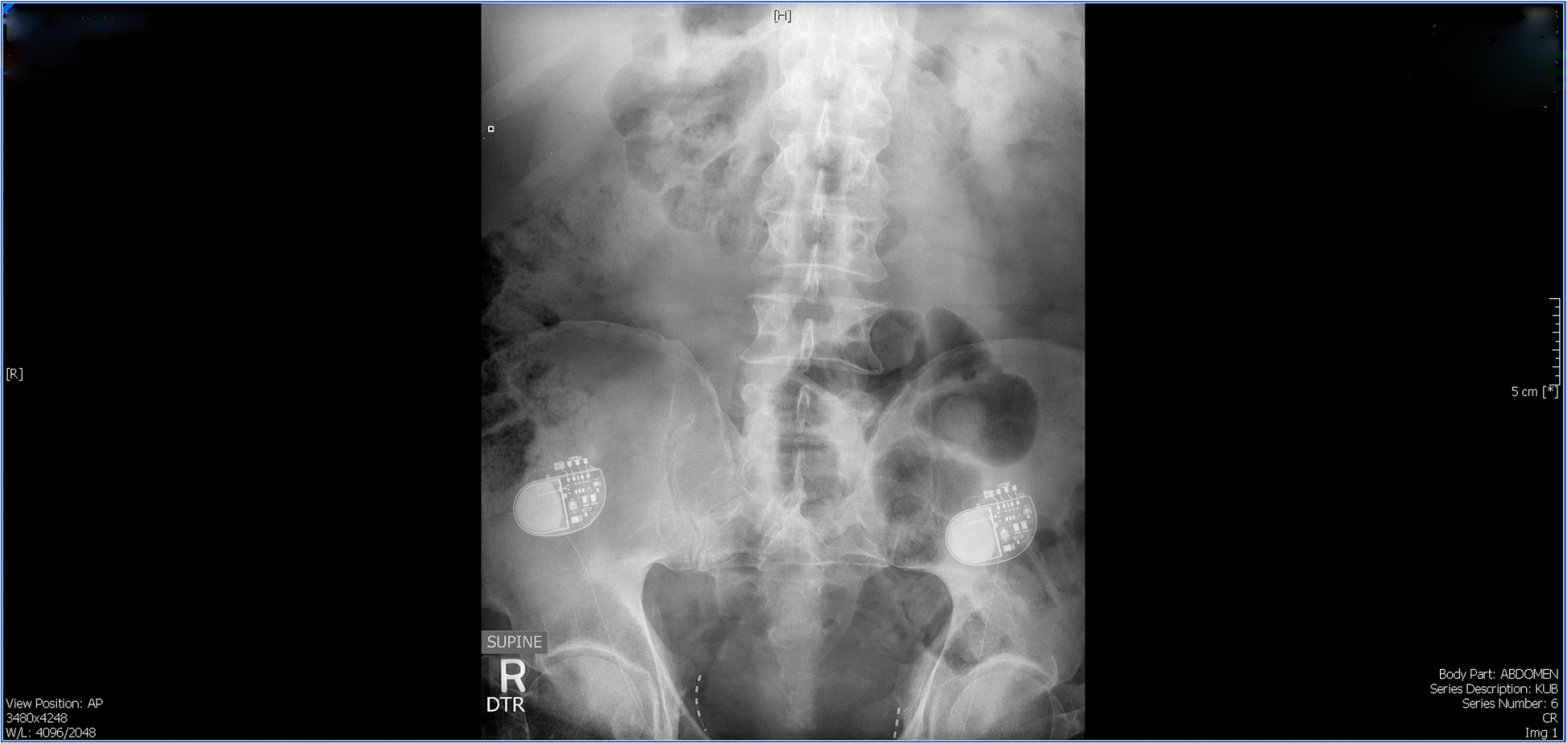
The written report of this patient’s first multichannel urodynamics study

M.E. is a 50-year-old Caucasian male, working as a Drum Dock Manager in a car manufacturing factory. On April 23, 2012, as he was unloading a forklift, the machine automatically engaged itself, speeding toward him and running him over. His quick-thinking co-workers positioned him flat on the floor with his head tilted to the left. M.E. was conscious and complaining of severe chest and pelvic pain. When emergency medical services arrived, he was stable. A decision was made to fly him to a nearby Trauma One Medical Center. He was diagnosed with seven left-sided rib fractures, transection of the pubic symphysis (Fig. 1), and L_1_-L_3_ transverse process fractures. There was blood at his urethral meatus, and after a retrograde urethrogram (RUG) with live fluoroscopy and without a hard copy print, a guide wire was placed through a cystoscope and into the bladder with fluoroscopic confirmation. M.E. later underwent orthopedic stabilization and repair of his pubis symphysis. His lumbar/sacral MRI redemonstrated the lumbar transverse fractures with moderate degenerative lumbar disc disease without nerve impingement (Fig. 2). The RUG demonstrated bulbar extravasation, but a Council tip catheter was successfully guided into the bladder to determine urethral continuity. At 6 weeks, his RUG demonstrated a well-healed urethra. He was given multiple trials of voids after being able to ambulate well for one month, but in spite of a sensation to void, he could not generate a detrusor pressure, and no voiding occurred. He failed two more trials of void. A urodynamics study was then performed (Fig. 3), demonstrating a first desire to void at 155 mls. Even at 550 mls, he experienced a strong desire to void but no detrusor pressure. The patient had good rectal sphincter tone and could volitionally tighten his rectum, so the pudendal nerve was assumed to be working well. We recommended a minimally invasive approach of sacral neuromodulation, which he successfully underwent 6 months after the accident. His bellows and ipsilateral plantar flexion were arrived at two volts on lead 0, 1 and 2, and at three volts on lead 3. Within six hours of surgery, he was emptying half his bladder, and post void residuals were 300 mls with straight catheterization. After 2 months of follow-up, he continued to urinate at maximum flows of 17.2 mls/sec but with 250–300 residual urines, so another sacral neuromodulation was performed on his contralateral side. He immediately improved to straight catheterization residual urine of 50–100 mls with a max flow of 16.8 mls/sec with 400 mls voided. Unfortunately 6 months later, after M.E. had returned to work, his uniform was irritating both of his implants, and within 3 weeks, he had a bilateral cellulitis with purulent drainage from both lead sites and implantable pulse generators. At surgery to remove each implants each buttock pocket wound culture grew non-methicillin resistant Staph aureus and he was subsequently treated with Ciprofloxin home intravenous therapy for six weeks and the resumption of clean intermittent catheterization. Three months later, re-examination revealed both sites to be well healed. Our next alternative was bilateral pudendal neuromodulation (Table 1), which would avoid placement near or cross over with the two formerly infected sites. Bilateral pudendal neuromodulation was performed with a right and left 0–3 lead result of 1,1,2,4, and 2,2,4,5 volts, and within six hours, the patient began to void without difficulty with a straight catheterization post-void residual urine of 0–50 mls and a max flow of 14.5 mls/sec. We then performed urodynamics, which demonstrated high pressure voiding with Pdet greater than 120 cm H2O. While having been on both tamsulosin and finesteride for more than 5 years, M.E. had failed medical therapy and was a high pressure voider. Sustained high pressure is well known to damage the bladder much like hypertension can damage the heart. To protect his bladder, we recommended a Green light transurethral resection of the prostate, which was performed on July 17, 2014. His subsequent straight catheterization post void residual urines had been 0–50 mls and his mean max flow was 22.6 mls/sec. With one-year of follow-up, his voiding parameters remain stable while his treatment power on both implantable pulse generators has been 2.8 volts on the right and 4.2 volts on the left. He feels perineal sensation and is without discomfort. We now follow the patient in 4-month intervals.

**Table 1:** Multichannel urodynamics report

1^st^ Sensation	165 mls
1^st^ Desire to Void	389 mls
Strong Desire to Void	510 mls
P_abd_	51 cm H_2_O
P_det_	0 cm H_2_O
Max Flow	0 ml/sec
Cough Leak Point Pressure	No leak
Rectal Tone	Contracts external sphincter around a finger in his rectum, indicating that the pudendal nerve is working well

## Discussion

Treatment of complete detrusor areflexia has included Valsalva voiding, intermittent straight catheterization, chronic Foley catheterization, and most recently sacral neuromodulation. Women achieve higher success rates than men with sacral neuromodulation, but the use of pudendal neuromodulation [2–8], particularly dual pudendal neuromodulation for the treatment of detrusor areflexia, has not been described. Several physicians with whom I have communicated have stated that pudendal neuromodulation for male urinary retention does not work well. How sacral and pudendal neuromodulation work to treat both overactive bladder symptoms and, to a lesser extent, urinary retention has not been clearly defined. What is important to note is that the lower the voltage level achieved to elicit good motor provocation during sacral neuromodulation (bellows and ipsilateral big toe plantar flexion and potentially pudendal neuromodulation anal wink with an EMG documented EMG tracing at less than or equal to 3 volts at one or more leads) the better the end result for urinary retention and overactive bladder symptoms. Our patient had both bilateral sacral neuromodulation and bilateral pudendal neuromodulation and feels that pudendal neuromodulation has given him the closest sensation and performance to his normal voiding prior to the 2012 accident. He has also noticed an improvement in erections. While he was unable to have any erections without a phosphodiesterase 5 inhibitor for two years prior to the accident, he now can manage an erection without any medication. His voiding max flow and post-void residuals are now normal for his age, and he checks his post void residual via a home ultrasound device provided free of charge for his use. Pudendal neuromodulation is used as a salvage surgical operation for those who, with sacral neuromodulation, experience a decrease in efficacy and now fail to meet treatment expectations. Pudendal neuromodulation has not been described for urinary retention, but with our patient, the procedure appears to have improved his outcome after volitional voiding was not previously possible and his sacral implants became infected. We hope that with this patient’s continued success, we can appreciate the potential utilization of pudendal neuromodulation when sacral neuromodulation has failed for urinary retention.

## Conclusion

Sacral neuromodulation may successfully correct traumatic urinary retention, in male patients. Additionally, pudendal neuromodulation can be successfully utilized as a salvage method for an infected sacral neuromodulation impulse generator (IPG), and tined lead with a return to proper voiding.

## Consent

Written informed consent was obtained from the patient was obtained from the patient for publication of this case report and any accompanying images. A copy of the written consent is available for review by the Editor of this journal.
